# *Lachnospiraceae* are emerging industrial biocatalysts and biotherapeutics

**DOI:** 10.3389/fbioe.2023.1324396

**Published:** 2024-01-04

**Authors:** Tom Zaplana, Solange Miele, Andrew C. Tolonen

**Affiliations:** Génomique Métabolique, Genoscope, Institut François Jacob, CEA, CNRS, University of Evry, Université Paris-Saclay, Evry, France

**Keywords:** *Lachnospiraceae*, microbial biotechnology, microbiome, biocatalyst, live biotherapeutics, Clostridia

## Abstract

The *Lachnospiraceae* is a family of anaerobic bacteria in the class Clostridia with potential to advance the bio-economy and intestinal therapeutics. Some species of *Lachnospiraceae* metabolize abundant, low-cost feedstocks such as lignocellulose and carbon dioxide into value-added chemicals. Others are among the dominant species of the human colon and animal rumen, where they ferment dietary fiber to promote healthy gut and immune function. Here, we summarize recent studies of the physiology, cultivation, and genetics of *Lachnospiraceae*, highlighting their wide substrate utilization and metabolic products with industrial applications. We examine studies of these bacteria as Live Biotherapeutic Products (LBPs), focusing on *in vivo* disease models and clinical studies using them to treat infection, inflammation, metabolic syndrome, and cancer. We discuss key research areas including elucidation of intra-specific diversity and genetic modification of candidate strains that will facilitate the exploitation of *Lachnospiraceae* in industry and medicine.

## Introduction

The *Lachnospiraceae*, from the ancient Greek “lachnos” (wooly hair) and “spira” (coil, twist), is a family of anaerobic, mesophilic bacteria with Gram-positive ultrastructure. *Lachnospiraceae*, which generally correspond to Clostridia cluster XIVa (Whitman, 2009), inhabit diverse ecosystems. Host-associated species are found in the gastrointestinal tracts of humans (Gosalbes et al., 2011), mice (Meehan and Beiko, 2014), insects (Vera-Ponce de Leon et al., 2022), and ruminants (Seshadri et al., 2018) as well as the human gingival crevice (Antezack et al., 2021). Metagenomic studies have shown *Lachnospiraceae* account for 10%–45% of the total bacteria in feces of healthy adults (Liu C. et al., 2021) and have a life-long association with humans; they colonize the guts of infants and are enriched in the fecal microbiomes of long-living (>90 years old) individuals (Kong et al., 2016). Other *Lachnospiraceae* live in anaerobic soil (Hengstmann et al., 1999) where they recycle plant matter and mediate biological soil disinfestation, a pesticide-free method to control soil-borne pathogens (Huang et al., 2019). *Lachnospiraceae* also inhabit aquatic sediments (Lomans et al., 2001; Dai et al., 2016), Antarctic green snow (Smirnova et al., 2021), wastewater (McLellan et al., 2013), and deep sea hydrothermal vents (Schouw et al., 2016).

Recent interest in the important roles of *Lachnospiraceae* in gut and environmental ecosystems has led to advances in the genomics, cultivation, and genetic manipulation of these bacteria. Here, we examine these advances and explore how they have set the stage for applying *Lachnospiraceae* in industrial and medical biotechnology. Many metagenomic studies comparing the fecal communities of healthy and diseased subjects have drawn associations between *Lachnospiraceae* and human health. However, associations from fecal metagenomics are sometimes difficult to interpret or conflicting, likely due to differences in study design and strain-specific differences, and have been reviewed previously (Vacca et al., 2020; Liu X. et al., 2021). Thus, we focus on insights gained from preclinical, rodent models and human clinical studies involving administration of live *Lachnospiraceae*. We discuss opportunities and current needs to develop *Lachnospiraceae* to produce value-added biochemicals from low-cost feedstocks and as live biotherapeutic products (LBPs).

## Phylogeny and genomes

**FIGURE 1:**
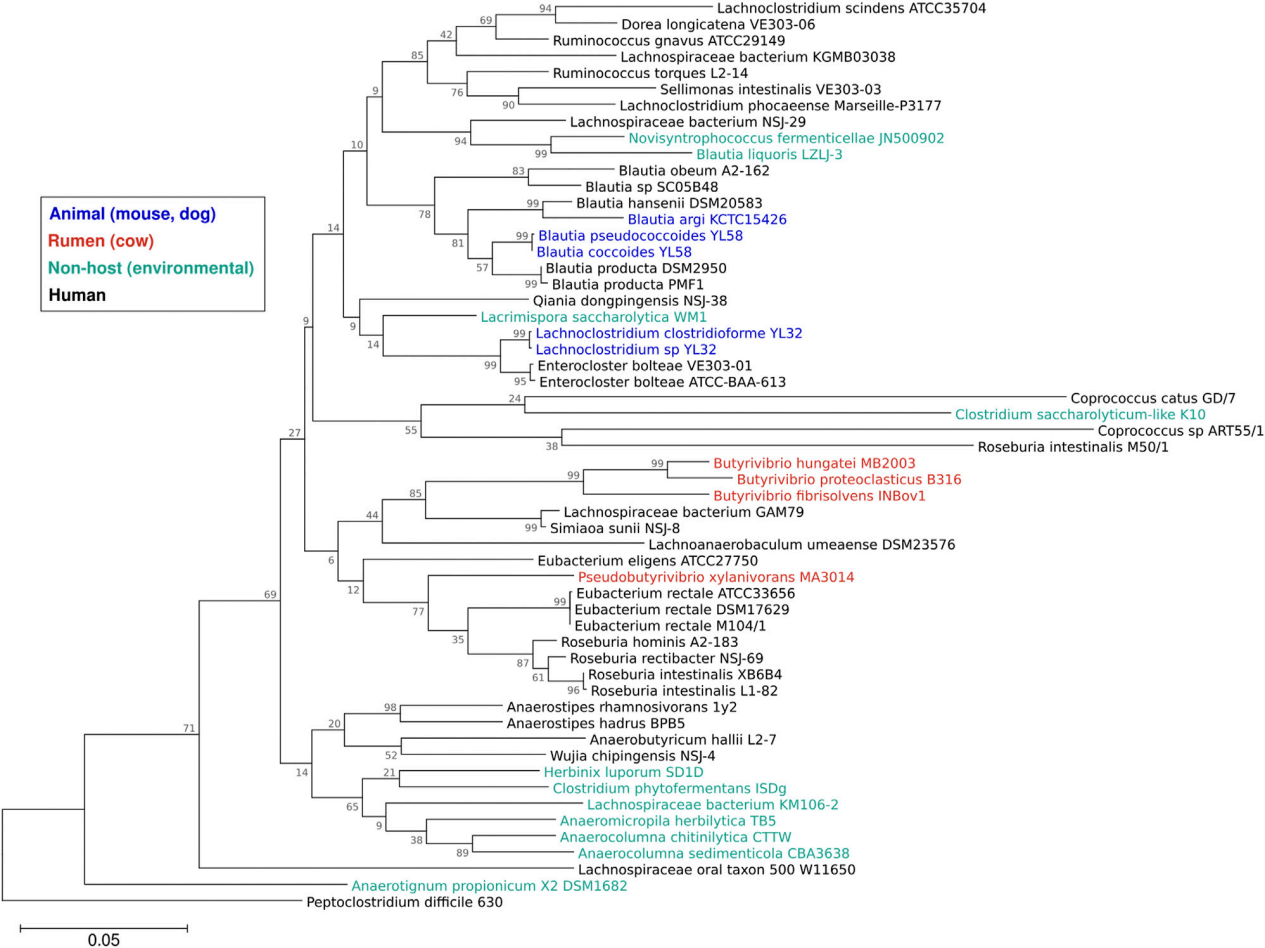
Phylogeny of the family *Lachnospiraceae*. Sequences of the 16S rRNA gene from the 57 *Lachnospiraceae* finished genomes in the IMG database were aligned using Muscle (Edgar, 2004) and a maximum likelihood tree (1,000 bootstraps) was constructed using Mega11 software (Tamura et al., 2021) with *Clostridioides (Peptoclostridium) difficile* 630 as the outgroup. Colors show strain isolation sites: animal (dog, mouse) feces (blue), cow rumen (red), human feces or oral cavity (black), or non-host environmental (aqua). Branch numbers are bootstrap percentages, branch lengths are substitution per site.

Our knowledge of *Lachnospiraceae* diversity and genomics has greatly expanded over the past decade. In 2014, the NCBI taxonomy of *Lachnospiraceae* included 24 genera (Meehan and Beiko, 2014), which by 2023 had increased to 118 genera with 1,941 species (NCBI Genome Datasets, 2023). The number of *Lachnospiraceae* genomes has similarly increased. In 2014, NCBI included 30 *Lachnospiraceae* genomes (Meehan and Beiko, 2014), which reached 201 genomes in 2023 (NCBI Genome Datasets, 2023). The Integrated Microbial Genomes & Microbiomes (IMG) portal (Chen I.-M. A. et al., 2023) includes 1,292 *Lachnospiraceae* genomic sequences, of which 57 are finished genomes. Most of the finished *Lachnospiraceae* genomes are of strains isolated from human feces or oral cavity, with strains from the cow rumen, animal feces, and environmental isolates forming clusters in the *Lachnospiraceae* phylogeny (Figure 1).

**FIGURE 2:**
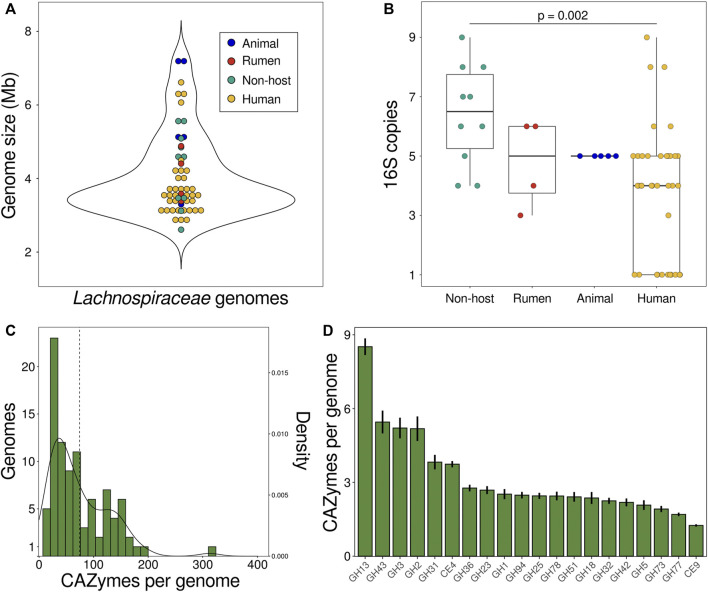
Characteristics of *Lachnospiraceae* genomes. **(A)** Genomes sizes and **(B)** number of copies of the 16S gene in 57 finished *Lachnospiraceae* genomes in the IMG database. Each data point is a genome and colors show strain isolation sites: animal (dog, mouse) feces (blue), cow rumen (red), human feces or oral cavity (yellow), or non-host environmental (aqua). **(C)** Number of CAZyme genes encoded by *Lachnospiraceae* genomes. The dashed line is the mean number of CAZymes per genome and the curve shows the kernel density estimation. **(D)** Number of CAZyme genes per genome for the 20 most abundant CAZyme families summed across *Lachnospiraceae* genomes. Bars show the mean ± SEM. Data in **(C,D)** is for degradative enzymes (glycoside hydrolases, carbohydrate esterases, pectin lyases) for 98 species in the CAZy database defined as *Lachnospiraceae* based on NCBI taxonomy. Mb DNA megabases. SEM standard error of the mean.

The GC content of *Lachnospiraceae* genomes varies between 35%–50%, with members of the same genus typically sharing similar GC levels (Sorbara et al., 2020). The distribution of *Lachnospiraceae* genome sizes (mean 4.06 Mb, median 3.59 Mb) is similar across habitats (Figure 2A). *Herbinix luporum* SD1D, a cellulose-degrading strain isolated from a biogas reactor, has the smallest genome of 2.6 Mb encoding 2,632 genes (Koeck et al., 2016). *Lachnoclostridium clostridioforme* YL32, from the mouse gut, has the largest genome of 7.2 Mb with 7,735 genes (Garzetti et al., 2017). Among these finished genomes, non-host species have a higher number of 16S genes than species isolated from humans (Figure 2B). Higher ribosomal gene copy number is linked to increased maximum growth rate and ability to respond to resource changes (Stevenson and Schmidt, 2004), suggesting these traits are under greater selection in non-host species.

## Substrate utilization

**FIGURE 3:**
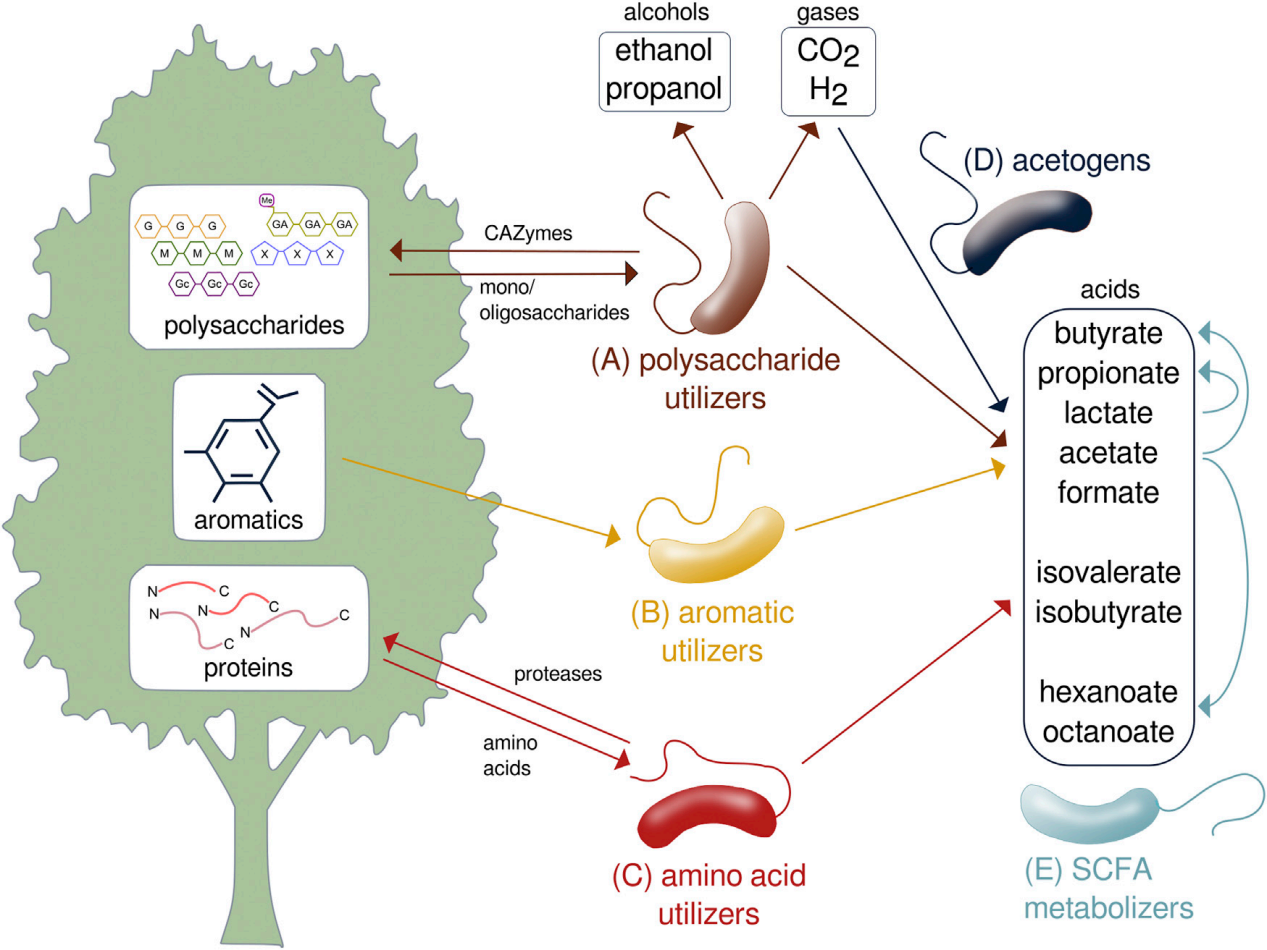
Metabolic transformation of lignocellulose performed by species of *Lachnospiraceae*. Specialized species metabolize the **(A)** polysaccharide, **(B)** aromatic, and **(C)** protein components of lignocellulose to produce gases, acids, and alcohols. **(D)** Acetogens transform carbon dioxide and hydrogen to SCFAs. **(E)** SCFAs are further transformed and assimilated by other species. SCFA, short-chain fatty acid; CO_2_, carbon dioxide; H_2_, hydrogen; CAZyme, carbohydrate-active enzyme.

Species of *Lachnospiraceae* collectively metabolize plant biomass through the assimilation of polysaccharides, peptides, and aromatics as well as subsequent transformation of the fermentation products by acetogens and cross-feeding species (Figure 3). As primary degraders of plant biomass, many *Lachnospiraceae* ferment a variety of complex polysaccharides including glucans, mannans, xylans, galactans, pectins, and arabinans (Boutard et al., 2014). Biomass-fermenting species sometimes grow faster on polysaccharides than on the constituent monosaccharides (Boutard et al., 2016). While typically able to ferment multiple polysaccharides, species of *Lachnospiraceae* are ecologically differentiated by specializing on certain substrates. As such, addition of different glycans results in compound-specific changes to the relative abundances of *Lachnospiraceae* in mixed communities (Tolonen et al., 2022). For example, *Roseburia intestinalis* efficiently metabolizes β-mannans and xylan (Leth et al., 2018; La Rosa et al., 2019), while *Roseburia faecis* ferments arabinogalactan (Sheridan et al., 2016). *Lachnospira* include pectin specialists, called pectinophiles (Cornick et al., 1994), that are stimulated by pectin addition (Bang et al., 2018). *Lachnospiraceae* specialized to metabolize cellulose are found in soil (Warnick et al., 2002; Wolin et al., 2003; Dai et al., 2016) and the rumen (Cai and Dong, 2010).

Genomes of plant-fermenting *Lachnospiraceae* encode numerous carbohydrate-active enzymes (CAZymes), each of which cleave a specific glycosidic linkage to depolymerize complex glycans. *Lachnospiraceae* genomes encode an average of 73 degradative CAZymes (glycoside hydrolases, pectin lyases, carbohydrate esterases) with less than 10% of genomes encoding more than 150 degradative CAZymes (Figure 2C). *Lachnospiraceae* bacterium CE91-St56, closely related to *Eisenbergiella massiliensis*, encodes over 100 more CAZymes than any other strain with 313 CAZymes including 294 glycoside hydrolases (Figure 2C). CE91-St56 was isolated from a supercentenarian (>110 years old) (Sato et al., 2021), highlighting the presence of *Lachnospiraceae* with extensive polysaccharide utilization capabilities in long-lived individuals. *Lachnospiraceae* genomes encode many families of CAZymes, the most abundant of which is GH13 to depolymerize α-glucans including starch and pullulan (Figure 2D). Other abundant CAZyme families enable degradation of substrates such as β-glucans (GH3,5,51,94), β-galactans (GH42), xylan (GH43), pectin (GH78), and chitin (GH18) (Figure 2D).

Some *Lachnospiraceae* assimilate C1 and C2 carbon sources that are produced during the initial stages of plant biomass fermentation. *Blautia hydrogenotrophica* is a human gut acetogen that grows autotrophically using hydrogen to fix carbon dioxide into acetyl-CoA by the Wood–Ljungdahl pathway (Bernalier et al., 1996) and other *Lachnospiraceae* acetogens consume H2 and CO_2_ in the cow rumen (Greening and Leedle, 1989). Species that assimilate other C1 and C2 molecules include *Sporobacterium*, isolated from an olive mill, that grows on methanol (Mechichi et al., 1999). Various *Lachnospiraceae* such as *Anaerobutyricum soehngenii* (previously *Eubacterium hallii*) metabolize acetate to butyrate (Udayappan et al., 2016; Zhang et al., 2019), which represents an important cross-feeding interaction in the gut (Flint et al., 2012).

Other *Lachnospiraceae* are specialized to metabolize peptides, alkanes, and aromatics. *Falcatimonas natans*, isolated from a methanogenic reactor, ferments peptides but not carbohydrates (Watanabe et al., 2016). *Abyssivirga alkaniphila*, a hydrothermal vent species, can grow on straight and branched alkanes (C5 to C25) using thiosulfate as an external electron acceptor (Schouw et al., 2016). *Syntrophococcus sucromutans* can oxidize sugars to acetate using formate or methoxymonobenzenoids as electron acceptors (Krumholz and Bryant, 1986). *Parasporobacterium* and *Sporobacterium* are soil bacteria that grow on methoxylated aromatic compounds as sole carbon and energy sources by catabolizing them to short-chain fatty acids (SCFAs) (Lomans et al., 2001). These aromatics are degraded by transforming the side chains and cleaving the aromatic ring using the phloroglucinol pathway (Whitman, 2009), as has recently been shown for *Clostridium* *scatologenes* (Zhou et al., 2023).

## Metabolic products

*Lachnospiraceae* metabolism yields alcohols, gases, and acids with importance in industry and human health (Table 1). Species producing industrially-relevant alcohols include *Lachnoclostridium phytofermentans*, which ferments cellulose to ethanol at 68% of the maximum theoretical yield (Tolonen et al., 2011) and, similar to *Roseburia inulinivorans*, metabolizes fucose and rhamnose to 1-propanol through a 1,2-propanediol intermediate in polyhedral microcompartments (Scott et al., 2006; Petit et al., 2013). *Lachnospiraceae* produce hydrogen by co-expressing monomeric and multimeric, bifurcating [FeFe] hydrogenases along with energy conserving [NiFe] hydrogenases (Calusinska et al., 2010) to enable yields reaching 2–3 moles of hydrogen per mole glucose (Harvey et al., 2008).

**TABLE 1:** Fermentation products of Lachnospiraceae.

Product	Input	Enzymatic steps (EC numbers)	Representative Species	References
Formic acid	Pyruvate	2.3.1.54	*Roseburia intestinalis*	Hillman et al. (2020)
Acetic acid	Acetyl-CoA	2.3.1.8, 2.7.2.1	*Fusicatenibacter saccharivorans*	Takada et al. (2013)
Propionic acid	Lactate (acrylate pathway)	2.8.3.1, 4.2.1.54, 1.3.1.84	*Coprococcus cactus*	Sheridan et al. (2022)
	L-fucose (propanediol pathway)	5.3.1.25, 2.7.1.51, 4.1.2.17, 1.1.1.77, 4.2.1.28, 1.2.1.87, 2.8.3.1	*Roseburia inulinivorans*	Scott et al. (2006)
	L-rhamnose (propanediol pathway)	5.3.1.14, 2.7.1.15, 4.1.2.19, 1.1.1.77, 4.2.1.28, 1.2.1.87, 2.8.3.1	*Lachnoclostridium phytofermentans*	Petit et al. (2013)
	3-oxopropionate (myo-inositol pathway)	2.8.3.5, 1.1.1.35, 4.2.1.116, 1.3.1.95, 2.8.3.1	*Anaerostipes rhamnosivorans*	Bui et al. (2021)
L-lactic acid	Pyruvate	1.1.1.27	*Lachnoclostridium phytofermentans*	Tolonen et al. (2011)
Butyric acid	Acetyl-CoA, (BCoAT)	2.3.1.9, 1.1.1.157, 4.2.1.55, 1.3.1.86, 2.8.3.8	*Roseburia intestinalis*	Vital et al. (2014)
	Acetyl-CoA (Butyrate kinase)	2.3.1.9, 1.1.1.157, 4.2.1.55, 1.3.1.86, 2.3.1.17, 2.7.2.7	*Eubacterium ventriosum*	Vital et al. (2014)
	Lysine	5.4.3.2, 5.4.3.3, 1.4.1.11, 2.3.1.247, 4.3.1.14, 1.3.1.86, 2.8.3.8	*Lachnospiraceae sp F0167*	Vital et al. (2014)
	Glutarate	1.1.99.2, 2.8.3.12, 4.2.1.167, 7.2.4.5, 1.3.1.86, 2.8.3.8	*Clostridiales* sp SS3/4	Vital et al. (2014)
	4-aminobutyrate	1.1.1.61, 2.8.3-, 4.2.1.120, 1.3.1.86, 2.8.3.8	*Anaerostipes caccae*	Vital et al. (2014)
Isobutyric acid	Valine	1.4.1.23/2.6.1.66, 4.1.1.72, 1.2.1.3	*Falcatimonas natans*	Watanabe et al. (2016)
Succinate	Pyruvate	2.7.1.40, 4.1.1.49, 1.1.1.37, 4.2.1.2, 1.3.5.1	*Blautia wexlerae*	Hosomi et al. (2022)
Isovaleric acid	Leucine	1.4.1.9/2.6.1.6, 4.1.1.72, 1.2.13	*Falcatimonas natans*	Watanabe et al. (2016)
5-aminovaleric acid	Proline	1.21.4.1	*Dorea longicatena*	Lopez et al. (2020)
Indoleacetic acid	Tryptophan	2.6.1.57, 4.1.1.74, 1.2.3.7	*Anaerobutyricum soehngenii*	Russell et al. (2013)
Hexanoic, octanoic acid	Acetyl-CoA	2.3.1.16, 1.1.1.35, 4.2.1.17, 1.3.8.7, 3.1.2.20	*Candidatus Weimeria bifida*	Scarborough et al. (2020)
Ethanol	Acetyl-CoA	1.2.1.10, 1.1.1.1	*Lachnoclostridium phytofermentans*	Tolonen et al. (2015b)
1-Propanol	L-fucose	5.3.1.25, 2.7.1.51, 4.1.2.17, 1.1.1.77, 4.2.1.28, 1.1.1.1	*Roseburia inulinivorans*	Scott et al. (2006)
	L-rhamnose	5.3.1.14, 2.7.1.15, 4.1.2.19, 1.1.1.77, 4.2.1.28, 1.1.1.1	*Lachnoclostridium phytofermentans*	Petit et al. (2013)
Hydrogen	Ferredoxin, H+	1.12.7.2	*Roseburia intestinalis*	Dostal et al. (2015)

Shown are the metabolic product, metabolite entering the pathway, enzymatic steps comprising the pathway, and a representative species containing the pathway with a supporting reference. EC number, Enzyme Commission number.

Fermentation of dietary fiber by gut *Lachnospiraceae* yields three main SCFA (acetate, propionate, and butyrate) (Table 1), all of which have been shown to benefit health. Acetate, which is synthesized in two steps from acetyl-CoA, reduces adipose accumulation and improves glucose tolerance (Yamashita et al., 2007). *Lachnospiraceae* use the acrylate, propanediol, and myo-inositol pathways to synthesize propionate (Reichardt et al., 2014; Bui et al., 2021), which is absorbed from the gut into the bloodstream to regulate cholesterol (Berggren et al., 1996) and reduce visceral and liver fat accumulation (Chambers et al., 2015). Propionate also enhances satiety (Arora et al., 2011), making it a potential way to reduce obesity. Along with *Ruminococcaceae*, *Lachnospiraceae* are the dominant butyrate producers in the human gut (Vital et al., 2014). While butyrate is most often synthesized from acetyl-CoA using butyryl-CoA transferase or butyrate kinase, some *Lachnospiraceae* can produce butyrate from lysine, glutarate, and 4-aminobutyrate (Vital et al., 2014), highlighting their abilities produce butyrate from different nutritional sources. Butyrate is the preferred energy source of colonocytes (Litvak et al., 2018), suppresses pathogens (Walker et al., 2021), and stimulates differentiation of anti-inflammatory T-regulatory cells (Furusawa et al., 2013).

Other organic acids produced by *Lachnospiraceae* include the branched-chain fatty acids isobutyrate and isovalerate that are synthesized from valine and leucine, respectively (Watanabe et al., 2016). *Candidatus* Weimeria bifida metabolizes pentoses to medium-chain fatty acids (hexanoate and octanoate) using the reverse β-oxidation cycle for SCFA chain elongation (Scarborough et al., 2020). Species of *Blautia* metabolize pyruvate to succinate (Hosomi et al., 2022) and, depending on the species, *Lachnospiraceae* can either produce L-lactate (Tolonen et al., 2011) or metabolize DL-lactate to acetate or butyrate using stereospecific lactate dehydrogenases (Sheridan et al., 2022). Gut *Lachnospiraceae* ferment proline to 5-aminovalerate in competition with *Clostridioides difficile*, representing a potential means to prevent colonization by this pathogen (Lopez et al., 2020). *Lachnospiraceae* ferment aromatic amino acids (tryptophan, tyrosine, phenylalanine) to phenolic and indolic acids (Russell et al., 2013), including compounds such as indoleacetic acid that promote intestinal homeostasis by signaling through the aryl hydrocarbon receptor (Roager and Licht, 2018).

*Lachnospiraceae* also produce other metabolites with health and industrial applications. *Lachnospiraceae* can modify and cleave the heterocyclic C-ring of flavonoids (Braune and Blaut, 2016) yielding molecules such as equol, which is linked to prevention of colorectal cancer (Sugiyama et al., 2013) and aging-related disorders (Mayo et al., 2019). Gut *Lachnospiraceae* generate reactive sulfur species that protect the host from oxidative stress-induced liver injury (Uchiyama et al., 2022). *Lachnospiraceae* produce farnesol (Abdugheni et al., 2022), an isoprene-derived molecule with anti-inflammatory and neuroprotective activities (Sell et al., 2022). Farnesol also has industrial applications as a fragrance ingredient (Lapczynski et al., 2008) and diesel fuel precursor (Rude and Schirmer, 2009). *Butyrivibrio fibrisolvens* synthesizes an exopolysaccharide with potential industrial applications that is rheologically similar to xanthan gum, but is composed of rare sugars including L-altrose and L-iduronic acid (Wachenheim and Patterson, 1992; Ferreira et al., 1997).

*Lachnospiraceae* are a potential source of other antimicrobial and immunomodulatory compounds. *Blautia obeum* synthesizes a lantibiotic, nisin O, that inhibits pathogens, including *C. difficile* and *Clostridium perfringens* (Hatziioanou et al., 2017). *Lachnospiraceae* produce pyrazines, which are being developed as antimicrobial and anti-fungal drugs (Hassan et al., 2020). Gut *Lachnospiraceae* convert primary bile acids by 7α-dehydroxylation to secondary bile acids that inhibit enteric pathogens and regulate mucosal immunity (Jin et al., 2022). Nonribosomal peptide synthetases (NRPS) of human gut *Lachnospiraceae* produce immunomodulatory secondary metabolites such as di-peptide aldehydes that act as cell-permeable cathepsin inhibitors, which could act as immunosuppressors by blocking antigen processing (Guo et al., 2017).

## Cultivation and engineering

*Lachnospiraceae* are mesophiles that grow at 30°C–45°C and can be cultivated under standard anaerobic conditions using jars with anaerobic sachets or a glove box. A human gut *Lachnospiraceae* biobank (hLchsp) was established as part of the China General Microorganism Culture Collection by isolating strains from healthy adult fecal samples, yielding a collection of 77 species across 33 genera with *Lachnospira*, *Blautia*, and *Roseburia* being the most abundant genera (Abdugheni et al., 2022). Among the seven growth media used for isolations, Yeast Casitone Fatty Acid (YCFA) medium supported the greatest number of *Lachnospiraceae* species, which comprised 19.6% of all bacterial isolates from the fecal samples. Another project to cultivate human gut *Lachnospiraceae* using rich, non-selective media yielded 273 isolates, including all the *Lachnospiraceae* genera that were detected by metagenomic sequences of the donor feces (Sorbara et al., 2020). In contrast to gut species, more oligotrophic, GS2 medium is used to cultivate environmental heterotrophic *Lachnospiraceae* such as *Herbinix hemicellulosilytica* and *L. phytofermentans* (Warnick et al., 2002; Koeck et al., 2015). The acetogen *B. hydrogenotrophica* can be grown on DMSZ 114 or general-acetogen (GA) medium, either as an autotroph using H_2_:CO_2_ or as a heterotroph by adding a carbon source (Groher and Weuster-Botz, 2016).

**FIGURE 4:**
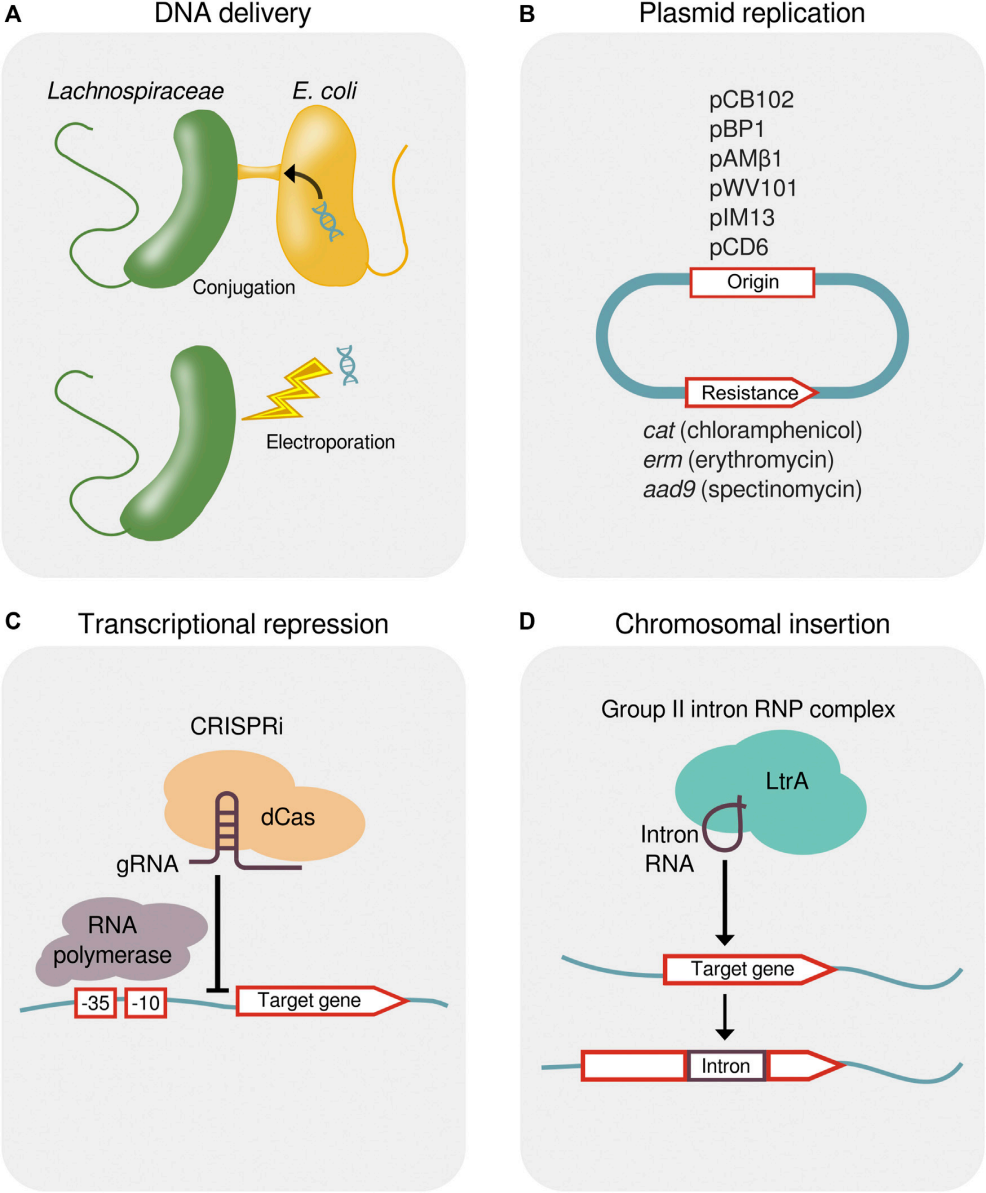
Methods for the genetic manipulation of *Lachnospiraceae*. **(A)** Delivery of foreign DNA into *Lachnospiraceae* by either conjugal transfer from *E. coli* or electroporation. **(B)** Plasmid origins that replicate in *Lachnospiraceae* and antibiotic resistance genes for plasmid selection. **(C)** Transcriptional repression by CRISPRi showing a dCas protein targeted by a gRNA to bind upstream of a gene, thereby blocking progression of RNA polymerase. **(D)** Targeted chromosomal insertion using a designed group II intron (targetron). Genomic insertion of the group II intron RNA containing a 13–16 bp target recognition sequence is facilitated by the endonuclease and reverse transcriptase activity of the LtrA protein. CRISPRi, CRISPR interference; dCas, dead CRISPR-associated protein; gRNA, guide RNA; RNP, ribonucleoprotein.

Methods for the genetic modification of *Lachnospiraceae* are being developed to study their molecular biology and to engineer optimized strains. Early studies found that conjugative transposons bearing antibiotic resistance genes transfer DNA between *Lachnospiraceae* (Barbosa et al., 1999). Experimental methods have been developed to transfer plasmid DNA into species of *Lachnoclostridium*, *Roseburia*, *Eubacterium*, *Enterocloster*, *Lacrimispora*, and *Blautia* by conjugation with *Escherichia coli* (Tolonen et al., 2009; Cuív et al., 2015; Sheridan et al., 2019; Jin et al., 2022) and by electroporation into species of *Lachnoclostridium* and *Butyrivibrio* (Beard et al., 1995; Rostain et al., 2022) (Figure 4A).

Native plasmids from *Lachnospiraceae* have been used as expression vectors (Hefford et al., 1997) and the pMTL plasmid system (Heap et al., 2009) can be applied to identify plasmid origins and resistance markers that function in strains of interest (Jin et al., 2022; Rostain et al., 2022). Plasmid origins that have been shown to replicate in *Lachnospiraceae* include pCB102 from *Clostridium butyricum*, pBP1 from *Clostridium botulinum*, pAMβ1 from *Enterococcus faecalis*, pWV101 from *Lactococcus lactis*, pIM13 from *Bacillus subtilis*, and pCD6 from *C. difficile*, and these plasmids can be maintained using antibiotic resistance genes *catP* (chloramphenicol), *ermB* (erythromycin), or *aad9* (spectinomycin) (Sheridan et al., 2019; Jin et al., 2022; Rostain et al., 2022) (Figure 4B).

Shuttle vectors containing Gram-negative and Gram-positive origins of replication have permitted the heterologous expression of a β-(1,3–1,4)-glucanase in *Eubacterium rectale* and *R. inulinivorans* (Sheridan et al., 2019) and of a synthetic ethanol formation pathway in *L. phytofermentans* (Tolonen et al., 2015b). Plasmid-based expression of a NanoLuc reporter has identified a library of promoters of varying strength, and reporter gene expression can be regulated by anhydrotetracycline using a promoter flanked tet repressor sites (Rostain et al., 2022). Clustered Regularly Interspaced Short Palindromic Repeats interference (CRISPRi) using dCas12a has been applied to repress the transcription of chromosomal genes for fermentation, thereby reducing production of butyrate in *Blautia luti* and *Enterocloster boltae* (Jin et al., 2022) and acetate in *L. phytofermentans* (Rostain et al., 2022) (Figure 4C).

Similar to other Clostridia, low rates of DNA transfer and homologous recombination in *Lachnospiraceae* have led to the use of other recombination systems to make targeted chromosomal changes. Designed group II intron called targetrons enabled gene inactivation by targeted chromosome insertion in various *Lachnospiraceae* with efficiencies ranging from 12.5%–100% (Tolonen et al., 2009; Tolonen et al., 2015a; Cerisy et al., 2019a; Jin et al., 2022) (Figure 4D). Multi-gene fragments can be excised and inserted by modifying targetrons to deliver *lox* sites into the genome that act as anchor points for Cre-mediated recombination, which has been applied to delete a 39 kb prophage in *L. phytofermentans* (Cerisy et al., 2019b).

*Lachnospiraceae* proteins that have been applied as molecular tools in other organisms are strong candidates to advance genome engineering in *Lachnospiraceae*. For example, LbCas12a, which was isolated from *Lachnospiraceae* bacterium ND 2006 (Tak et al., 2017), has been applied for genome editing in eukaryotes including fungi (Chen T. et al., 2023), plants (Kim et al., 2021), flies (Port et al., 2020), and human cells (Zhang et al., 2023). Another Cas12a variant, Lb2Cas12a, derived from *Lachnospiraceae* bacterium MA 2020, has been developed to have enhanced editing activity and broadened protospacer adjacent motif (PAM) recognition in human cells (Tran et al., 2021). A protein with anti-CRISPR function has been identified from *Lachnospiraceae* phage (Forsberg et al., 2019) and a cytosine deaminase from *R. intestinalis* has been fused to CRISPR and Transcription Activator-Like Effector (TALE) proteins to make targeted C-to-T transitions in the genomes of cultured cells and mouse embryos (Guo et al., 2023).

## Bioeconomy applications

The capabilities of *Lachnospiraceae* to metabolize the polysaccharides, aromatics, and proteins that compose lignocellulose make them candidates to transform low-cost, sustainable, lignocellulosic feedstocks (i.e., forestry, agricultural, and municipal wastes) into value-added biochemicals (Table 1). For example, *L. phytofermentans*, a species with wide polysaccharide utilization capabilities, ferments corn stover to ethanol with efficiencies similar to commercial enzymes and xylose-fermenting yeast (Jin et al., 2011). Synthesis of longer chain acids from lignocellulose residues by *Ca.* Weimeria bifida (Scarborough et al., 2020) holds potential to develop ‘drop in’ fuels that are compatible with the current petroleum infrastructure. *F. natans* metabolizes the protein fraction of organic matter to produce isobutyrate and n-butyrate, which are used for artificial fibers, plastics and herbicides as well as isovalerate used for flavoring and perfumes (Agnihotri et al., 2022).

*Lachnospiraceae* have been identified as biocatalysts for conversion of biomass to hydrogen (Bu et al., 2021), and strains have been isolated that produce 2–3 moles hydrogen per mole of glucose equivalent (Harvey et al., 2008). In addition to its use as a fuel, hydrogen produced from lignocellulosic fermentation can be used as a reductant to fix CO and CO_2_ by acetogens through gas fermentation (Figure 3), a process that has potential to convert industrial carbon emissions to useful biochemicals (Liew et al., 2016). *Lachnospiraceae* acetogens compete with methanogens in the cow rumen, representing a means to reduce bovine methane production (Yang et al., 2015).

Alternatively, hydrogen from lignocellulosic fermentation can be used to fix carbon dioxide by methanogens, and *Lachnospiraceae* have been shown to actuate the transformation of cellulose to methane by methanogenic consortia (Dai et al., 2016). Combination of *Lachnospiraceae* with other microorganisms to form synthetic consortia has been generally used to enhanced product formation rates (Zuroff et al., 2013; El Hage et al., 2019; Park et al., 2020), and engineered consortia including biomass-fermenting *Lachnospiraceae* and methanogens or acetogens have potential for the direct conversion of lignocellulose to methane and other value-added biochemicals.

## Therapeutic applications: preclinical models

Preclinical *in vivo* models are providing evidence that supplementation with live *Lachnospiraceae* improves gut health and prevents pathogen colonization. For example, in a rat model of irritable bowel syndrome, addition of *Roseburia hominis* increased cecal butyrate content, reduced visceral hypersensitivity, and prevented the decreased expression of occludin (Zhang et al., 2019). Administration of *Blautia producta* directly inhibited growth of vancomycin-resistant *Enterococcus* (Caballero et al., 2017) and a murine *Lachnospiraceae* inhibits *C. difficile* colonization in mice (Reeves et al., 2012).

*Lachnospiraceae* can alleviate inflammatory and allergic diseases by modulating the immune system through production of antigens presented by innate immune cells and immunomodulatory metabolites. *R. intestinalis* promoted differentiation of regulatory T cells, activation of type 3 innate lymphoid cells, and suppression of inflammation through TLR5 (Shen et al., 2022). *E. rectale* supplementation regulated dendritic cell activation by reducing the frequency of CD83^+^ cells and improved symptoms in a mouse model of Behçet’s disease, a systemic inflammatory condition (Islam et al., 2021). Inoculation of germ-free mice with a 17 strain consortium including 8 *Lachnospiraceae* increased anti-inflammatory, regulatory T-cells (CD4^+^, FoxP3+) in the colonic lamina propria of multiple mouse lines and alleviated colitis in experimental models (Atarashi et al., 2013). In addition, administration of this 17 strain consortium to mice in an ovalbumin (OVA)-induced allergic diarrhea mouse model reduced diarrhea and OVA-specific serum IgE levels (Atarashi et al., 2013). Additional evidence that *Lachnospiraceae* can mitigate food allergies was demonstrated by colonization of germ-free mice with *Anaerostipes caccae*, which protected against an anaphylactic response upon challenge with β-Lactoglobulin (BLG), a cow’s milk allergen, and reduced BLG-specific IgE levels (Feehley et al., 2019).

*Lachnospiraceae* have provided benefits in preclinical models of metabolic syndrome and diabetes. Administration of *Blautia wexlerae* to male C57BL/6 mice on a high fat diet reduced body weight and multiple diabetes indicators, which was linked to *B. wexlerae* metabolites such as S-adenosylmethionine, acetylcholine, and L-ornithine conferring anti-adipogenesis and anti-inflammatory properties to adipocytes (Hosomi et al., 2022). *B. producta* suppressed lipid accumulation in HepG2 cells and gavage of *B. producta* alleviated hyperlipidemia in mice through production of 12-methylmyristic acid (Wu et al., 2021). Treatment of obese, diabetic *db/db* mice with *A. soehngenii* reduced plasma glucose, epididymal fat, and liver triglycerides and improved peripheral insulin sensitivity (Udayappan et al., 2016).

Mouse studies have also shown beneficial roles of *Lachnospiraceae* in cancer treatment. C57BL/6 mice bearing B16-F10 melanoma or CT-26 colorectal tumors showed reduced tumor growth when administered *Blautia massiliensis* (Goodman et al., 2019). Anti-PD1 mediated tumor control and survivorship of C57BL/6 mice bearing B16-F10 melanoma tumors was improved by administration of *E. rectale*, which was proposed to result from it consuming L-serine, leading to NK cell activation and tumor infiltration (Liu et al., 2023). *E. rectale* treatment also reduced the incidence of lymphoma and reduced TNF levels in sensitized Eμ-Myc mice (Lu et al., 2022). Survivorship and clinical scores of C57BL/6 mice following full body irradiation were increased by prior inoculation with a mix of 23 *Lachnospiraceae*, which resulted from increased hematopoiesis and reduced intestinal epithelial injury (Guo et al., 2020).

## Therapeutic applications: clinical trials

*Lachnospiraceae* are being tested as LBPs in clinical studies for a number of diseases (Table 2), the most advanced of which is the treatment of recurrent *C. difficile* infections (rCDI). In an exploratory study of two rCDI patients at Queen’s University, treatment with a synthetic community of 33 strains, including 10 *Lachnospiraceae*, resulted in full remission in both patients (Petrof et al., 2013). Subsequently, microbial consortia containing *Lachnospiraceae* have been developed to combat rCDI. SER-109, consisting of spores purified from fecal samples, is composed of 77 genera of which 36% are *Lachnospiraceae* (Feuerstadt et al., 2022). In a phase 3 study, SER-109 reduced rCDI at week 8 (Feuerstadt et al., 2022). In 2023, the US Food and Drug Administration approved SER-109 under the commercial name Vowst^TM^ to treat recurrent *C. difficile* infection (Commission of the US FDA, 2023). VE303, a consortium of 8 strains including 5 *Lachnospiraceae*, engrafted into the microbiomes of healthy volunteers to boost production of SCFA and secondary bile acids without any serious adverse events (Dsouza et al., 2022). Phase 2 results in patients supported that rCDI at 8 weeks was reduced by VE303 (Louie et al., 2023). VE303 is being tested in a phase 3 study to treat rCDI starting in 2023, and as an experimental treatment for hepatic encephalopathy (Vedanta Biosciences Inc, 2023).

**TABLE 2:** Clinical studies of Lachnospiraceae as live biotherapeutic products.

Principal Investigator; Sponsor	Trial	Intervention	Patient population	Outcome	Trial number, reference
Dr. Elaine Petrof, Queen’s University	Open label trial	Synthetic community	rCDI	Both subjects clinically cured at 6 months	NCT01372943 (Petrof et al., 2013)
Dr. Lisa von Moltke, Seres Therapeutics	Phase 3, double-blind, placebo-controlled	SER-109	rCDI	Reduced rCDI at week 8 (*p* < 0.001)	NCT03183128 (Feuerstadt et al., 2022)
Dr. Michele Trucksis, Seres Therapeutics	Phase 2 double-blind, placebo-controlled	SER-109	rCDI	Reduced rCDI (44.1% vs. 53.3% with placebo), but not significant. Engraftment associated with non-recurrence (*p* < 0.05) and increased secondary bile acid concentrations (*p* < 0.0001)	NCT02437487 (McGovern et al., 2022)
Seres Therapeutics	Phase 1b Safety Study	SER-287	Mild to moderate UC	Increased clinical remission at week 8 (*p* = 0.024)	NCT02618187 (Henn et al., 2021)
Dr Eamonn Quigley, Houston Methodist; 4DPharma	Phase 2 double-blind, placebo-controlled	MRx1234 (Blautix)	IBS	Improved bowel habits (*p* = 0.007) and trend to increased overall response (*p* = 0.06)	NCT03721107 (Quigley et al., 2023)
Dr. Darrell Pardi, Mayo Clinic; Vedanta Biosciences	Phase 2 double-blind, placebo-controlled	VE303	rCDI	Reduced rCDI at week 8 (*p* = 0.006)	NCT03788434 (Louie et al., 2023)
Vedanta Biosciences	Phase 1 safety study	VE303	Healthy adults	Well tolerated, engraftment	NCT04236778 (Dsouza et al., 2022)
Dr. Patricia Bloom, University of Michigan; Vedanta Biosciences	Double-blind, placebo-controlled	VE303	Hepatic encephalopathy	Trial in progress	NCT04899115
Vedanta Biosciences	Phase 2, double-blind, placebo-controlled	VE202	Mild to moderate UC	Trial in progress	NCT05370885
Vedanta Biosciences	Phase 1 safety study	VE202	Healthy adults	Well tolerated, strain engraftment	Silber et al. (2022)
Dr. Erik Stroes, University of Amsterdam; Caelus Pharmaceuticals	Phase 2, double-blind placebo controlled	*Anaerobutyricum soehngenii* L2-7	Metabolic Syndrome	Elevated plasma GLP-1 (*p* = 0.02), increased fecal butyrate (*p* = 0.06), reduced glucose variability (*p* = 0.05)	NTR-NL6630, (Koopen et al., 2022)
Dr. James Ryan Atlantia Food Clinical Trials; Caelus Pharmaceuticals	Phase 1/2 dose finding study	*Anaerobutyricum soehngenii* L2-7	Metabolic Syndrome	Abundance of *A. soehngenii* correlated with peripheral insulin sensitivity (*p* = 0.05)	NCT04529473 (Gilijamse et al., 2020)
Oluf B Pedersen, University of Copenhagen	Placebo-controlled crossover study	*Ruminococcus torques* strain ATCC 27756	Overweight adults	Trial in progress	NCT05448274

The synthetic community in the Queen’s University study consisted of 33 strains, including 10 *Lachnospiraceae*. SER-109 is purified fecal spores, 36% of genera are *Lachnospiraceae*. SER-287 is purified fecal spores, 44% of genera are *Lachnospiraceae*. MRx1234 (Blautix) is lyophilised *Blautia hydrogenotrophica*. VE303 is a consortium of 8 strains including 5 *Lachnospiraceae*. VE202 is a consortium of 16 *Clostridia* XIVa, IV, XVIII. rCDI, recurrent *Clostridioides difficile* infection; UC, ulcerative colitis; IBS, irritable bowel syndrome.

LBPs that include *Lachnospiraceae* are also being developed for metabolic syndrome and inflammatory bowel disease (Table 2). For example, *A. soehngenii* L2-7 is being tested as a probiotic to improve insulin sensitivity in metabolic syndrome patients. A phase 1/2 study of 24 metabolic syndrome patients correlated *A. soehngenii* engraftment with improved peripheral insulin sensitivity (Gilijamse et al., 2020). A phase 2 study of 12 metabolic syndrome patients showed duodenal infusion of *A. soehngenii* reduced glucose variability and elevated GLP-1, secondary bile acids in plasma, duodenal REGB1 expression, and fecal SCFAs (Koopen et al., 2022). As *B. hydrogenotrophica* consumes intestinal gas (H_2_, CO_2_), it can treat irritable bowel syndrome (IBS) by reducing intestinal bloating. A phase 2 study of MRx1234 (Blautix), consisting of lyophilized *B. hydrogenotrophica*, improved bowel habits in IBS patients (Quigley et al., 2023).

## Looking forward: challenges and opportunities

*Lachnospiraceae* have numerous potential applications due to their native fermentation of low-cost substrates and importance for intestinal health, but key challenges remain to harness them as industrial biocatalysts and LBPs. In particular, work is needed to isolate and characterize additional *Lachnospiraceae* strains, uncover the genetic basis of their physiological traits, and, ultimately, apply these learning to engineer optimized strains. Recently established *Lachnospiraceae* collections demonstrated methods with which many species can be cultivated (Seshadri et al., 2018; Sorbara et al., 2020; Abdugheni et al., 2022). Additional cultivation efforts could build *Lachnospiraceae* collections from environments such as soil and human patient populations. Further, *Lachnospiraceae* culture collections revealed that isolates from the same species can differ in traits such as substrate utilization and production of antimicrobials (Sorbara et al., 2020). Intraspecific comparative genomics of isolates sharing similar genomes, but with specific physiological differences, is an opportunity to define genotype-phenotype relationships in *Lachnospiraceae*.

Development of microorganisms for biotechnology often requires rewiring native metabolism to improve product yields, highlighting the importance of future research on genetic manipulation of *Lachnospiraceae*. As described above, genetic tools have been ported to *Lachnospiraceae* from other well-studied mesophiles including *B. subtilis* and *L. lactis*. Species of *Lachnospiraceae* have been established as genetically tractable hosts with methods for genetic transformation, plasmid replication and selection, transcriptional repression, and chromosomal insertions (Figure 4); a recent study demonstrates that these methods can be generally applied to many species (Jin et al., 2022). Existing molecular tools derived from *Lachnospiraceae* are promising candidates to advance genome engineering in these bacteria, including Cas proteins with broadened PAM recognition (Tran et al., 2021), anti-CRISPR proteins (Forsberg et al., 2019), and cytosine deaminases (Guo et al., 2023). Moreover, adaptive laboratory evolution can be used to generate *Lachnospiraceae* strains with complex, multigenic traits such as inhibitor tolerance that are intractable by rational genome engineering (Cerisy et al., 2017).

Engineering of *Lachnospiraceae* for industrial production of biochemicals from low-cost feedstocks will need to focus on substrate assimilation and product formation. The rate of lignocellulose solubilization remains a primary obstacle to its utilization as a feedstock (Preethi et al., 2021), which could be addressed by engineering strains with modified expression of CAZymes and associated ABC transporters to accelerate solubilization and uptake of target lignocellulosic substrates. As *Lachnospiraceae* typically produce a mixture of fermentation products, redistribution of metabolic flux by repression or inactivation of genes for undesired products is a valuable approach to streamline and increase fermentation yields.

Genetic manipulation of *Lachnospiraceae* will also be important to define the mechanisms by which they promote intestinal health. Significant advances have been made to elucidate innate and adaptive immune responses modulated by *Lachnospiraceae* (Islam et al., 2021, 1) (Atarashi et al., 2013; Feehley et al., 2019; Liu et al., 2023). It is generally believed that *Lachnospiraceae* modulate host immunity producing SCFAs. Recently, it was shown that differential recognition of *Lachnospiraceae* flagellins by TLR5 contributes to immune tolerance (Clasen et al., 2023). Genetic studies with *Lachnospiraceae* mutants will be useful to further define the mechanisms that underlie host interactions and build strains with customized immunomodulatory properties.

Industrial-scale cultivation of *Lachnospiraceae* for bioproduction and LBPs will necessitate a greater understanding of the genetics and ecology of the phage that infect them. Phage infection is recognized as a persistent threat in industrial microbiology where scale-up of bacterial populations in large bioreactors favors phage outbreaks. For example, phage infection is the main cause of fermentation failures in the dairy industry, leading to intense study of the phage of lactic acid bacteria (Fernández et al., 2017). Prophages are common in the genomes of gut *Lachnospiraceae* (Dikareva et al., 2023). Although hypervirulent phage can drive temporal variation of *R. intestinalis* abundances in the intestine, no phage infecting *Lachnospiraceae* have been deposited in public databases (Cornuault et al., 2020). Thus, isolation and characterization of *Lachnospiraceae* phage are important subjects for future research.

Over the past decade, our understanding of *Lachnospiraceae* molecular biology and physiology has greatly increased. In 2023, Vowst^TM^ became the first FDA-approved oral medication containing live *Lachnospiraceae* (Commission of the US FDA, 2023) and clinical studies are evaluating additional therapeutic benefits of *Lachnospiraceae* (Table 2). Development of optimized strains by genome engineering will enable us to realize the potential of *Lachnospiraceae* in biotechnology. In the next few years, we expect to see further development of *Lachnospiraceae* to produce useful chemicals and to manage disease through targeted changes to the composition and metabolites produced by the gut microbiome.

## References

[B1] Abdugheni R. Wang W.-Z. Wang Y.-J. Du M.-X. Liu F.-L. Zhou N. (2022). Metabolite profiling of human-originated Lachnospiraceae at the strain level. iMeta 1, e58. 10.1002/imt2.58 PMC10989990 38867908

[B2] Agnihotri S. Yin D.-M. Mahboubi A. Sapmaz T. Varjani S. Qiao W. (2022). A glimpse of the world of volatile fatty acids production and application: a review. Bioengineered 13, 1249–1275. 10.1080/21655979.2021.1996044 34738864 PMC8805862

[B3] Antezack A. Boxberger M. La Scola B. Monnet-Corti V. (2021). Isolation and description of catonella massiliensis sp. nov., a novel catonella species, isolated from a stable periodontitis subject. Pathogens 10, 367. 10.3390/pathogens10030367 33808593 PMC8003473

[B4] Arora T. Sharma R. Frost G. (2011). Propionate. Anti-obesity and satiety enhancing factor? Appetite 56, 511–515. 10.1016/j.appet.2011.01.016 21255628

[B5] Atarashi K. Tanoue T. Oshima K. Suda W. Nagano Y. Nishikawa H. (2013). Treg induction by a rationally selected mixture of Clostridia strains from the human microbiota. Nature 500, 232–236. 10.1038/nature12331 23842501

[B6] Bang S.-J. Kim G. Lim M. Y. Song E.-J. Jung D.-H. Kum J.-S. (2018). The influence of *in vitro* pectin fermentation on the human fecal microbiome. Amb. Express 8, 98. 10.1186/s13568-018-0629-9 29909506 PMC6004267

[B7] Barbosa T. M. Scott K. P. Flint H. J. (1999). Evidence for recent intergeneric transfer of a new tetracycline resistance gene, tet(W), isolated from Butyrivibrio fibrisolvens, and the occurrence of tet(O) in ruminal bacteria. Environ. Microbiol. 1, 53–64. 10.1046/j.1462-2920.1999.00004.x 11207718

[B8] Beard C. E. Hefford M. A. Forster R. J. Sontakke S. Teather R. M. Gregg K. (1995). A stable and efficient transformation system for Butyrivibrio fibrisolvens OB156. Curr. Microbiol. 30, 105–109. 10.1007/BF00294191 7765886

[B9] Berggren A. M. Nyman E. M. Lundquist I. Björck I. M. (1996). Influence of orally and rectally administered propionate on cholesterol and glucose metabolism in obese rats. Br. J. Nutr. 76, 287–294. 10.1079/bjn19960032 8813902

[B10] Bernalier A. Willems A. Leclerc M. Rochet V. Collins M. D. (1996). Ruminococcus hydrogenotrophicus sp. nov., a new H2/CO2-utilizing acetogenic bacterium isolated from human feces. Arch. Microbiol. 166, 176–183. 10.1007/s002030050373 8703194

[B11] Boutard M. Cerisy T. Nogue P.-Y. Alberti A. Weissenbach J. Salanoubat M. (2014). Functional diversity of carbohydrate-active enzymes enabling a bacterium to ferment plant biomass. PLoS Genet. 10, e1004773. 10.1371/journal.pgen.1004773 25393313 PMC4230839

[B12] Boutard M. Ettwiller L. Cerisy T. Alberti A. Labadie K. Salanoubat M. (2016). Global repositioning of transcription start sites in a plant-fermenting bacterium. Nat. Commun. 7, 13783. 10.1038/ncomms13783 27982035 PMC5171806

[B13] Braune A. Blaut M. (2016). Bacterial species involved in the conversion of dietary flavonoids in the human gut. Gut Microbes 7, 216–234. 10.1080/19490976.2016.1158395 26963713 PMC4939924

[B14] Bu J. Wei H.-L. Wang Y.-T. Cheng J.-R. Zhu M.-J. (2021). Biochar boosts dark fermentative H2 production from sugarcane bagasse by selective enrichment/colonization of functional bacteria and enhancing extracellular electron transfer. Water Res. 202, 117440. 10.1016/j.watres.2021.117440 34304072

[B15] Bui T. P. N. Mannerås-Holm L. Puschmann R. Wu H. Troise A. D. Nijsse B. (2021). Conversion of dietary inositol into propionate and acetate by commensal Anaerostipes associates with host health. Nat. Commun. 12, 4798. 10.1038/s41467-021-25081-w 34376656 PMC8355322

[B16] Caballero S. Kim S. Carter R. A. Leiner I. M. Sušac B. Miller L. (2017). Cooperating commensals restore colonization resistance to vancomycin-resistant Enterococcus faecium. Cell Host Microbe 21, 592–602.e4. 10.1016/j.chom.2017.04.002 28494240 PMC5494988

[B17] Cai S. Dong X. (2010). Cellulosilyticum ruminicola gen. nov., sp. nov., isolated from the rumen of yak, and reclassification of Clostridium lentocellum as Cellulosilyticum lentocellum comb. nov. Int. J. Syst. Evol. Microbiol. 60, 845–849. 10.1099/ijs.0.014712-0 19661493

[B18] Calusinska M. Happe T. Joris B. Wilmotte A. (2010). The surprising diversity of clostridial hydrogenases: a comparative genomic perspective. Microbiol. Read. 156, 1575–1588. 10.1099/mic.0.032771-0 20395274

[B19] Cerisy T. Iglesias A. Rostain W. Boutard M. Pelle C. Perret A. (2019a). ABC transporters required for hexose uptake by Clostridium phytofermentans. J. Bacteriol. 201, e00241-19. 10.1128/JB.00241-19 31109990 PMC6620405

[B20] Cerisy T. Rostain W. Chhun A. Boutard M. Salanoubat M. Tolonen A. C. (2019b). A targetron-recombinase system for large-scale genome engineering of clostridia. mSphere 4, e00710-19. 10.1128/mSphere.00710-19 PMC6908422 31826971

[B21] Cerisy T. Souterre T. Torres-Romero I. Boutard M. Dubois I. Patrouix J. (2017). Evolution of a biomass-fermenting bacterium to resist lignin phenolics. Appl. Environ. Microbiol. 83, e00289-17. 10.1128/AEM.00289-17 28363966 PMC5440714

[B22] Chambers E. S. Viardot A. Psichas A. Morrison D. J. Murphy K. G. Zac-Varghese S. E. K. (2015). Effects of targeted delivery of propionate to the human colon on appetite regulation, body weight maintenance and adiposity in overweight adults. Gut 64, 1744–1754. 10.1136/gutjnl-2014-307913 25500202 PMC4680171

[B23] Chen I.-M. A. Chu K. Palaniappan K. Ratner A. Huang J. Huntemann M. (2023a). The IMG/M data management and analysis system v.7: content updates and new features. Nucleic Acids Res. 51, D723–D732. 10.1093/nar/gkac976 36382399 PMC9825475

[B24] Chen T. Chen Z. Zhang H. Li Y. Yao L. Zeng B. (2023b). Development of a CRISPR/Cpf1 system for multiplex gene editing in Aspergillus oryzae. Folia Microbiol. (Praha). 10.1007/s12223-023-01081-9 37490214

[B25] Clasen S. J. Bell M. E. W. Borbón A. Lee D.-H. Henseler Z. M. de la Cuesta-Zuluaga J. (2023). Silent recognition of flagellins from human gut commensal bacteria by Toll-like receptor 5. Sci. Immunol. 8, eabq7001. 10.1126/sciimmunol.abq7001 36608151

[B26] Commission of the US Food and Drug Administration (2023). FDA approves first orally administered fecal microbiota product for the prevention of recurrence of Clostridioides difficile infection. https://www.fda.gov/news-events/press-announcements/fda-approves-first-orally-administered-fecal-microbiota-product-prevention-recurrence-clostridioides.

[B27] Cornick N. A. Jensen N. S. Stahl D. A. Hartman P. A. Allison M. J. (1994). Lachnospira pectinoschiza sp. nov., an anaerobic pectinophile from the pig intestine. Int. J. Syst. Bacteriol. 44, 87–93. 10.1099/00207713-44-1-87 8123565

[B28] Cornuault J. K. Moncaut E. Loux V. Mathieu A. Sokol H. Petit M.-A. (2020). The enemy from within: a prophage of Roseburia intestinalis systematically turns lytic in the mouse gut, driving bacterial adaptation by CRISPR spacer acquisition. ISME J. 14, 771–787. 10.1038/s41396-019-0566-x 31827247 PMC7031369

[B29] Cuív P. Ó. Smith W. J. Pottenger S. Burman S. Shanahan E. R. Morrison M. (2015). Isolation of genetically tractable most-wanted bacteria by metaparental mating. Sci. Rep. 5, 13282. 10.1038/srep13282 26293474 PMC4642544

[B30] Dai Y. Yan Z. Jia L. Zhang S. Gao L. Wei X. (2016). The composition, localization and function of low-temperature-adapted microbial communities involved in methanogenic degradations of cellulose and chitin from Qinghai-Tibetan Plateau wetland soils. J. Appl. Microbiol. 121, 163–176. 10.1111/jam.13164 27123875

[B31] Dikareva E. Matharu D. Lahtinen E. Kolho K.-L. De Vos W. M. Salonen A. (2023). An extended catalog of integrated prophages in the infant and adult fecal microbiome shows high prevalence of lysogeny. Front. Microbiol. 14, 1254535. 10.3389/fmicb.2023.1254535 37731926 PMC10508911

[B32] Dostal A. Lacroix C. Bircher L. Pham V. T. Follador R. Zimmermann M. B. (2015). Iron modulates butyrate production by a child gut microbiota *in vitro* . mBio 6, e01453–e01415. 10.1128/mBio.01453-15 26578675 PMC4659462

[B33] Dsouza M. Menon R. Crossette E. Bhattarai S. K. Schneider J. Kim Y.-G. (2022). Colonization of the live biotherapeutic product VE303 and modulation of the microbiota and metabolites in healthy volunteers. Cell Host Microbe 30, 583–598.e8. 10.1016/j.chom.2022.03.016 35421353

[B34] Edgar R. C. (2004). MUSCLE: a multiple sequence alignment method with reduced time and space complexity. BMC Bioinforma. 5, 113. 10.1186/1471-2105-5-113 PMC517706 15318951

[B35] El Hage R. Hernandez-Sanabria E. Calatayud Arroyo M. Props R. Van de Wiele T. (2019). Propionate-producing consortium restores antibiotic-induced dysbiosis in a dynamic *in vitro* model of the human intestinal microbial ecosystem. Front. Microbiol. 10, 1206. 10.3389/fmicb.2019.01206 31214145 PMC6554338

[B36] Feehley T. Plunkett C. H. Bao R. Choi Hong S. M. Culleen E. Belda-Ferre P. (2019). Healthy infants harbor intestinal bacteria that protect against food allergy. Nat. Med. 25, 448–453. 10.1038/s41591-018-0324-z 30643289 PMC6408964

[B37] Fernández L. Escobedo S. Gutiérrez D. Portilla S. Martínez B. García P. (2017). Bacteriophages in the dairy environment: from enemies to allies. Antibiotics 6, 27. 10.3390/antibiotics6040027 29117107 PMC5745470

[B38] Ferreira F. Kenne L. Cotta M. A. Stack R. J. (1997). Structural studies of the extracellular polysaccharide from Butyrivibrio fibrisolvens strain CF3. Carbohydr. Res. 301, 193–203. 10.1016/s0008-6215(97)00097-9 9232840

[B39] Feuerstadt P. Louie T. J. Lashner B. Wang E. E. L. Diao L. Bryant J. A. (2022). SER-109, an oral microbiome therapy for recurrent Clostridioides difficile infection. N. Engl. J. Med. 386, 220–229. 10.1056/NEJMoa2106516 35045228

[B40] Flint H. J. Scott K. P. Duncan S. H. Louis P. Forano E. (2012). Microbial degradation of complex carbohydrates in the gut. Gut Microbes 3, 289–306. 10.4161/gmic.19897 22572875 PMC3463488

[B41] Forsberg K. J. Bhatt I. V. Schmidtke D. T. Javanmardi K. Dillard K. E. Stoddard B. L. (2019). Functional metagenomics-guided discovery of potent Cas9 inhibitors in the human microbiome. eLife 8, e46540. 10.7554/eLife.46540 31502535 PMC6739867

[B42] Furusawa Y. Obata Y. Fukuda S. Endo T. A. Nakato G. Takahashi D. (2013). Commensal microbe-derived butyrate induces the differentiation of colonic regulatory T cells. Nature 504, 446–450. 10.1038/nature12721 24226770

[B43] Garzetti D. Brugiroux S. Bunk B. Pukall R. McCoy K. D. Macpherson A. J. (2017). High-quality whole-genome sequences of the oligo-mouse-microbiota bacterial community. Genome Announc. 5, 007588-17. 10.1128/genomeA.00758-17 PMC5646386 29051233

[B44] Gilijamse P. W. Hartstra A. V. Levin E. Wortelboer K. Serlie M. J. Ackermans M. T. (2020). Treatment with Anaerobutyricum soehngenii: a pilot study of safety and dose-response effects on glucose metabolism in human subjects with metabolic syndrome. NPJ Biofilms Microbiomes 6, 16. 10.1038/s41522-020-0127-0 32221294 PMC7101376

[B45] Goodman B. Sandy P. Papkoff J. Gardner H. Ponichtera H. (2019). Treating cancer using a blautia strain. US Patent application 2019/0240267 A1.

[B46] Gosalbes M. J. Durbán A. Pignatelli M. Abellan J. J. Jiménez-Hernández N. Pérez-Cobas A. E. (2011). Metatranscriptomic approach to analyze the functional human gut microbiota. PLOS ONE 6, e17447. 10.1371/journal.pone.0017447 21408168 PMC3050895

[B47] Greening R. C. Leedle J. A. Z. (1989). Enrichment and isolation of Acetitomaculum ruminis, gen. nov., sp. nov.: acetogenic bacteria from the bovine rumen. Arch. Microbiol. 151, 399–406. 10.1007/BF00416597 2500921

[B48] Groher A. Weuster-Botz D. (2016). General medium for the autotrophic cultivation of acetogens. Bioprocess Biosyst. Eng. 39, 1645–1650. 10.1007/s00449-016-1634-5 27270418

[B49] Guo C.-J. Chang F.-Y. Wyche T. P. Backus K. M. Acker T. M. Funabashi M. (2017). Discovery of reactive microbiota-derived metabolites that inhibit host proteases. Cell 168, 517–526.e18. 10.1016/j.cell.2016.12.021 28111075 PMC5302092

[B50] Guo H. Chou W.-C. Lai Y. Liang K. Tam J. W. Brickey W. J. (2020). Multi-omics analyses of radiation survivors identify radioprotective microbes and metabolites. Science 370, eaay9097. 10.1126/science.aay9097 33122357 PMC7898465

[B51] Guo J. Yu W. Li M. Chen H. Liu J. Xue X. (2023). A DddA ortholog-based and transactivator-assisted nuclear and mitochondrial cytosine base editors with expanded target compatibility. Mol. Cell 83, 1710–1724.e7. 10.1016/j.molcel.2023.04.012 37141888

[B52] Harvey S. Chambers A. Zhang P. (2008). Overproduction of hydrogen from an anaerobic bacterium. https://apps.dtic.mil/sti/citations/ADA505872.

[B53] Hassan N. W. Saudi M. N. Abdel-Ghany Y. S. Ismail A. Elzahhar P. A. Sriram D. (2020). Novel pyrazine based anti-tubercular agents: design, synthesis, biological evaluation and *in silico* studies. Bioorg Chem. 96, 103610. 10.1016/j.bioorg.2020.103610 32028062

[B54] Hatziioanou D. Gherghisan-Filip C. Saalbach G. Horn N. Wegmann U. Duncan S. H. (2017). Discovery of a novel lantibiotic nisin O from Blautia obeum A2-162, isolated from the human gastrointestinal tract. Microbiol. Read. 163, 1292–1305. 10.1099/mic.0.000515 PMC5882112 28857034

[B55] Heap J. T. Pennington O. J. Cartman S. T. Minton N. P. (2009). A modular system for Clostridium shuttle plasmids. J. Microbiol. Methods 78, 79–85. 10.1016/j.mimet.2009.05.004 19445976

[B56] Hefford M. A. Kobayashi Y. Allard S. E. Forster R. J. Teather R. M. (1997). Sequence analysis and characterization of pOM1, a small cryptic plasmid from Butyrivibrio fibrisolvens, and its use in construction of a new family of cloning vectors for Butyrivibrios. Appl. Environ. Microbiol. 63, 1701–1711. 10.1128/aem.63.5.1701-1711.1997 9143105 PMC168465

[B57] Hengstmann U. Chin K. J. Janssen P. H. Liesack W. (1999). Comparative phylogenetic assignment of environmental sequences of genes encoding 16S rRNA and numerically abundant culturable bacteria from an anoxic rice paddy soil. Appl. Environ. Microbiol. 65, 5050–5058. 10.1128/AEM.65.11.5050-5058.1999 10543822 PMC91680

[B58] Henn M. R. O’Brien E. J. Diao L. Feagan B. G. Sandborn W. J. Huttenhower C. (2021). A phase 1b safety study of SER-287, a spore-based microbiome therapeutic, for active mild to moderate ulcerative colitis. Gastroenterology 160, 115–127.e30. 10.1053/j.gastro.2020.07.048 32763240 PMC7402096

[B59] Hillman E. T. Kozik A. J. Hooker C. A. Burnett J. L. Heo Y. Kiesel V. A. (2020). Comparative genomics of the genus Roseburia reveals divergent biosynthetic pathways that may influence colonic competition among species. Microb. Genom 6, mgen000399. 10.1099/mgen.0.000399 32589566 PMC7478625

[B60] Hosomi K. Saito M. Park J. Murakami H. Shibata N. Ando M. (2022). Oral administration of Blautia wexlerae ameliorates obesity and type 2 diabetes via metabolic remodeling of the gut microbiota. Nat. Commun. 13, 4477. 10.1038/s41467-022-32015-7 35982037 PMC9388534

[B61] Huang X. Liu L. Zhao J. Zhang J. Cai Z. (2019). The families Ruminococcaceae, Lachnospiraceae, and Clostridiaceae are the dominant bacterial groups during reductive soil disinfestation with incorporated plant residues. Appl. Soil Ecol. 135, 65–72. 10.1016/j.apsoil.2018.11.011

[B62] Islam S. M. S. Ryu H.-M. Sayeed H. M. Byun H.-O. Jung J.-Y. Kim H.-A. (2021). Eubacterium rectale attenuates HSV-1 induced systemic inflammation in mice by inhibiting CD83. Front. Immunol. 12, 712312. 10.3389/fimmu.2021.712312 34531862 PMC8438521

[B63] Jin M. Balan V. Gunawan C. Dale B. E. (2011). Consolidated bioprocessing (CBP) performance of Clostridium phytofermentans on AFEX-treated corn stover for ethanol production. Biotechnol. Bioeng. 108, 1290–1297. 10.1002/bit.23059 21280028

[B64] Jin W.-B. Li T.-T. Huo D. Qu S. Li X. V. Arifuzzaman M. (2022). Genetic manipulation of gut microbes enables single-gene interrogation in a complex microbiome. Cell 185, 547–562.e22. 10.1016/j.cell.2021.12.035 35051369 PMC8919858

[B65] Kim D. Hager M. Brant E. Budak H. (2021). Efficient genome editing in wheat using Cas9 and Cpf1 (AsCpf1 and LbCpf1) nucleases. Funct. Integr. Genomics 21, 355–366. 10.1007/s10142-021-00782-z 33710467

[B66] Koeck D. E. Ludwig W. Wanner G. Zverlov V. V. Liebl W. Schwarz W. H. (2015). Herbinix hemicellulosilytica gen. nov., sp. nov., a thermophilic cellulose-degrading bacterium isolated from a thermophilic biogas reactor. Int. J. Syst. Evol. Microbiol. 65, 2365–2371. 10.1099/ijs.0.000264 25872956

[B67] Koeck D. E. Maus I. Wibberg D. Winkler A. Zverlov V. V. Liebl W. (2016). Complete genome sequence of Herbinix luporum SD1D, a new cellulose-degrading bacterium isolated from a thermophilic biogas reactor. Genome Announc. 4, 006877-16. 10.1128/genomeA.00687-16 PMC4956452 27445379

[B68] Kong F. Hua Y. Zeng B. Ning R. Li Y. Zhao J. (2016). Gut microbiota signatures of longevity. Curr. Biol. 26, R832–R833. 10.1016/j.cub.2016.08.015 27676296

[B69] Koopen A. Witjes J. Wortelboer K. Majait S. Prodan A. Levin E. (2022). Duodenal Anaerobutyricum soehngenii infusion stimulates GLP-1 production, ameliorates glycaemic control and beneficially shapes the duodenal transcriptome in metabolic syndrome subjects: a randomised double-blind placebo-controlled cross-over study. Gut 71, 1577–1587. 10.1136/gutjnl-2020-323297 34697034 PMC9279853

[B70] Krumholz L. R. Bryant M. P. (1986). Syntrophococcus sucromutans sp. nov. gen. nov. uses carbohydrates as electron donors and formate, methoxymonobenzenoids or Methanobrevibacter as electron acceptor systems. Arch. Microbiol. 143, 313–318. 10.1007/BF00412795

[B71] Lapczynski A. Bhatia S. P. Letizia C. S. Api A. M. (2008). Fragrance material review on farnesol. Food Chem. Toxicol. 46 (Suppl. 11), S149–S156. 10.1016/j.fct.2008.06.046 18640198

[B72] La Rosa S. L. Leth M. L. Michalak L. Hansen M. E. Pudlo N. A. Glowacki R. (2019). The human gut Firmicute Roseburia intestinalis is a primary degrader of dietary β-mannans. Nat. Commun. 10, 905. 10.1038/s41467-019-08812-y 30796211 PMC6385246

[B73] Leth M. L. Ejby M. Workman C. Ewald D. A. Pedersen S. S. Sternberg C. (2018). Differential bacterial capture and transport preferences facilitate co-growth on dietary xylan in the human gut. Nat. Microbiol. 3, 570–580. 10.1038/s41564-018-0132-8 29610517

[B74] Liew F. Martin M. E. Tappel R. C. Heijstra B. D. Mihalcea C. Köpke M. (2016). Gas fermentation—a flexible platform for commercial scale production of low-carbon-fuels and chemicals from waste and renewable feedstocks. Front. Microbiol. 7, 694. 10.3389/fmicb.2016.00694 27242719 PMC4862988

[B75] Litvak Y. Byndloss M. X. Bäumler A. J. (2018). Colonocyte metabolism shapes the gut microbiota. Science 362, eaat9076. 10.1126/science.aat9076 30498100 PMC6296223

[B76] Liu C. Du M.-X. Abuduaini R. Yu H.-Y. Li D.-H. Wang Y.-J. (2021a). Enlightening the taxonomy darkness of human gut microbiomes with a cultured biobank. Microbiome 9, 119. 10.1186/s40168-021-01064-3 34020714 PMC8140505

[B77] Liu N. Chen L. Yan M. Tao Q. Wu J. Chen J. (2023). Eubacterium rectale improves the efficacy of anti-PD1 immunotherapy in melanoma via l-serine-mediated NK cell activation. Research 6, 0127. 10.34133/research.0127 37223471 PMC10202379

[B78] Liu X. Mao B. Gu J. Wu J. Cui S. Wang G. (2021b). Blautia-a new functional genus with potential probiotic properties? Gut Microbes 13, 1–21. 10.1080/19490976.2021.1875796 PMC7872077 33525961

[B79] Lomans B. P. Leijdekkers P. Wesselink J. J. Bakkes P. Pol A. van der Drift C. (2001). Obligate sulfide-dependent degradation of methoxylated aromatic compounds and formation of methanethiol and dimethyl sulfide by a freshwater sediment isolate, Parasporobacterium paucivorans gen. nov., sp. nov. Appl. Environ. Microbiol. 67, 4017–4023. 10.1128/AEM.67.9.4017-4023.2001 11525999 PMC93123

[B80] Lopez C. A. McNeely T. P. Nurmakova K. Beavers W. N. Skaar E. P. (2020). Clostridioides difficile proline fermentation in response to commensal clostridia. Anaerobe 63, 102210. 10.1016/j.anaerobe.2020.102210 32422411 PMC8025294

[B81] Louie T. Golan Y. Khanna S. Bobilev D. Erpelding N. Fratazzi C. (2023). VE303, a defined bacterial consortium, for prevention of recurrent Clostridioides difficile infection: a randomized clinical trial. Jama 329, 1356–1366. 10.1001/jama.2023.4314 37060545 PMC10105904

[B82] Lu H. Xu X. Fu D. Gu Y. Fan R. Yi H. (2022). Butyrate-producing Eubacterium rectale suppresses lymphomagenesis by alleviating the TNF-induced TLR4/MyD88/NF-κB axis. Cell Host Microbe 30, 1139–1150.e7. 10.1016/j.chom.2022.07.003 35952646

[B83] Mayo B. Vázquez L. Flórez A. B. (2019). Equol: a bacterial metabolite from the daidzein isoflavone and its presumed beneficial health effects. Nutrients 11, 2231. 10.3390/nu11092231 31527435 PMC6770660

[B84] McGovern B. H. Ford C. B. Henn M. R. Pardi D. S. Khanna S. Hohmann E. L. (2021). SER-109, an investigational microbiome drug to reduce recurrence after Clostridioides difficile infection: lessons learned from a phase 2 trial. Clin. Infect. Dis. 72, 2132–2140. 10.1093/cid/ciaa387 32255488 PMC8204772

[B85] McLellan S. L. Newton R. J. Vandewalle J. L. Shanks O. C. Huse S. M. Eren A. M. (2013). Sewage reflects the distribution of human faecal *Lachnospiraceae* . Environ. Microbiol. 15, 2213–2227. 10.1111/1462-2920.12092 23438335 PMC4043349

[B86] Mechichi T. Labat M. Garcia J. L. Thomas P. Patel B. K. (1999). Sporobacterium olearium gen. nov., sp. nov., a new methanethiol-producing bacterium that degrades aromatic compounds, isolated from an olive mill wastewater treatment digester. Int. J. Syst. Bacteriol. 49 (Pt 4), 1741–1748. 10.1099/00207713-49-4-1741 10555356

[B87] Meehan C. J. Beiko R. G. (2014). A phylogenomic view of ecological specialization in the Lachnospiraceae, a family of digestive tract-associated bacteria. Genome Biol. Evol. 6, 703–713. 10.1093/gbe/evu050 24625961 PMC3971600

[B88] NCBI Genome Datasets (Lachnospiraceae) (2023). Genome, https://www.ncbi.nlm.nih.gov/datasets/genome/?taxon=186803&reference_only=true.

[B89] O Sheridan P. Martin J. C. Lawley T. D. Browne H. P. Harris H. M. B. Bernalier-Donadille A. (2016). Polysaccharide utilization loci and nutritional specialization in a dominant group of butyrate-producing human colonic Firmicutes. Microb. Genom 2, e000043. 10.1099/mgen.0.000043 28348841 PMC5320581

[B90] Park H. Patel A. Hunt K. A. Henson M. A. Carlson R. P. (2020). Artificial consortium demonstrates emergent properties of enhanced cellulosic-sugar degradation and biofuel synthesis. NPJ Biofilms Microbiomes 6, 59. 10.1038/s41522-020-00170-8 33268782 PMC7710750

[B91] Petit E. LaTouf W. G. Coppi M. V. Warnick T. A. Currie D. Romashko I. (2013). Involvement of a bacterial microcompartment in the metabolism of fucose and rhamnose by Clostridium phytofermentans. PLoS ONE 8, e54337. 10.1371/journal.pone.0054337 23382892 PMC3557285

[B92] Petrof E. O. Gloor G. B. Vanner S. J. Weese S. J. Carter D. Daigneault M. C. (2013). Stool substitute transplant therapy for the eradication of *Clostridium difficile* infection: ‘RePOOPulating’ the gut. Microbiome 1, 3. 10.1186/2049-2618-1-3 24467987 PMC3869191

[B93] Port F. Starostecka M. Boutros M. (2020). Multiplexed conditional genome editing with Cas12a in Drosophila. Proc. Natl. Acad. Sci. U. S. A. 117, 22890–22899. 10.1073/pnas.2004655117 32843348 PMC7502738

[B94] Preethi M. Kumar G. Karthikeyan O. P. Varjani S. (2021). Lignocellulosic biomass as an optimistic feedstock for the production of biofuels as valuable energy source: techno-economic analysis, Environmental Impact Analysis, Breakthrough and Perspectives. Environ. Technol. Innovation 24, 102080. 10.1016/j.eti.2021.102080

[B95] Quigley E. M. M. Markinson L. Stevenson A. Treasure F. P. Lacy B. E. (2023). Randomised clinical trial: efficacy and safety of the live biotherapeutic product MRx1234 in patients with irritable bowel syndrome. Aliment. Pharmacol. Ther. 57, 81–93. 10.1111/apt.17310 36369645

[B96] Reeves A. E. Koenigsknecht M. J. Bergin I. L. Young V. B. (2012). Suppression of *Clostridium difficile* in the gastrointestinal tracts of germfree mice inoculated with a murine isolate from the family Lachnospiraceae. Infect. Immun. 80, 3786–3794. 10.1128/IAI.00647-12 22890996 PMC3486043

[B97] Reichardt N. Duncan S. H. Young P. Belenguer A. McWilliam Leitch C. Scott K. P. (2014). Phylogenetic distribution of three pathways for propionate production within the human gut microbiota. ISME J. 8, 1323–1335. 10.1038/ismej.2014.14 24553467 PMC4030238

[B98] Roager H. M. Licht T. R. (2018). Microbial tryptophan catabolites in health and disease. Nat. Commun. 9, 3294. 10.1038/s41467-018-05470-4 30120222 PMC6098093

[B99] Rostain W. Zaplana T. Boutard M. Baum C. Tabuteau S. Sanitha M. (2022). Tuning of gene expression in Clostridium phytofermentans using synthetic promoters and CRISPRi. ACS Synth. Biol. 11, 4077–4088. 10.1021/acssynbio.2c00385 36427328 PMC9765743

[B100] Rude M. A. Schirmer A. (2009). New microbial fuels: a biotech perspective. Curr. Opin. Microbiol. 12, 274–281. 10.1016/j.mib.2009.04.004 19447673

[B101] Russell W. R. Duncan S. H. Scobbie L. Duncan G. Cantlay L. Calder A. G. (2013). Major phenylpropanoid-derived metabolites in the human gut can arise from microbial fermentation of protein. Mol. Nutr. Food Res. 57, 523–535. 10.1002/mnfr.201200594 23349065

[B102] Sato Y. Atarashi K. Plichta D. R. Arai Y. Sasajima S. Kearney S. M. (2021). Novel bile acid biosynthetic pathways are enriched in the microbiome of centenarians. Nature 599, 458–464. 10.1038/s41586-021-03832-5 34325466

[B103] Scarborough M. J. Myers K. S. Donohue T. J. Noguera D. R. (2020). Medium-chain fatty acid synthesis by “Candidatus Weimeria bifida” gen. Nov., sp. nov., and “Candidatus pseudoramibacter fermentans” sp. nov. Appl. Environ. Microbiol. 86, 022422-19. 10.1128/AEM.02242-19 PMC6974650 31704684

[B104] Schouw A. Leiknes Eide T. Stokke R. Pedersen R. B. Steen I. H. Bødtker G. (2016). Abyssivirga alkaniphila gen. nov., sp. nov., an alkane-degrading, anaerobic bacterium from a deep-sea hydrothermal vent system, and emended descriptions of Natranaerovirga pectinivora and Natranaerovirga hydrolytica. Int. J. Syst. Evol. Microbiol. 66, 1724–1734. 10.1099/ijsem.0.000934 26822139

[B105] Scott K. P. Martin J. C. Campbell G. Mayer C.-D. Flint H. J. (2006). Whole-genome transcription profiling reveals genes up-regulated by growth on fucose in the human gut bacterium “Roseburia inulinivorans.”. J. Bacteriol. 188, 4340–4349. 10.1128/JB.00137-06 16740940 PMC1482943

[B106] Sell L. B. Ramelow C. C. Kohl H. M. Hoffman K. Bains J. K. Doyle W. J. (2022). Farnesol induces protection against murine CNS inflammatory demyelination and modifies gut microbiome. Clin. Immunol. 235, 108766. 10.1016/j.clim.2021.108766 34091018 PMC8660955

[B107] Seshadri R. Leahy S. C. Attwood G. T. Teh K. H. Lambie S. C. Cookson A. L. (2018). Cultivation and sequencing of rumen microbiome members from the Hungate1000 Collection. Nat. Biotechnol. 36, 359–367. 10.1038/nbt.4110 29553575 PMC6118326

[B108] Shen Z. Luo W. Tan B. Nie K. Deng M. Wu S. (2022). Roseburia intestinalis stimulates TLR5-dependent intestinal immunity against Crohn’s disease. eBioMedicine 85, 104285. 10.1016/j.ebiom.2022.104285 36182776 PMC9526137

[B109] Sheridan P. O. Louis P. Tsompanidou E. Shaw S. Harmsen H. J. Duncan S. H. (2022). Distribution, organization and expression of genes concerned with anaerobic lactate utilization in human intestinal bacteria. Microb. Genom 8, 000739. 10.1099/mgen.0.000739 35077342 PMC8914356

[B110] Sheridan P. O. Martin J. C. Minton N. P. Flint H. J. O’Toole P. W. Scott K. P. (2019). Heterologous gene expression in the human gut bacteria Eubacterium rectale and Roseburia inulinivorans by means of conjugative plasmids. Anaerobe 59, 131–140. 10.1016/j.anaerobe.2019.06.008 31228669

[B111] Silber J. Norman J. Kanno T. Crossette E. Szabady R. Menon R. (2022). Randomized, double-blind, placebo (pbo)-controlled, single- and multiple-dose phase 1 study of ve202, a defined bacterial consortium for treatment of ibd: safety and colonization dynamics of a novel live biotherapeutic product (lbp) in healthy adults. Inflamm. Bowel Dis. 28, S65–S66. 10.1093/ibd/izac015.106 41342324

[B112] Smirnova M. Miamin U. Kohler A. Valentovich L. Akhremchuk A. Sidarenka A. (2021). Isolation and characterization of fast-growing green snow bacteria from coastal East Antarctica. MicrobiologyOpen 10, e1152. 10.1002/mbo3.1152 33377317 PMC7887010

[B113] Sorbara M. T. Littmann E. R. Fontana E. Moody T. U. Kohout C. E. Gjonbalaj M. (2020). Functional and genomic variation between human-derived isolates of Lachnospiraceae reveals inter- and intra-species diversity. Cell Host Microbe 28, 134–146.e4. 10.1016/j.chom.2020.05.005 32492369 PMC7351604

[B114] Stevenson B. S. Schmidt T. M. (2004). Life history implications of rRNA gene copy number in *Escherichia coli* . Appl. Environ. Microbiol. 70, 6670–6677. 10.1128/AEM.70.11.6670-6677.2004 15528533 PMC525164

[B115] Sugiyama Y. Masumori N. Fukuta F. Yoneta A. Hida T. Yamashita T. (2013). Influence of isoflavone intake and equol-producing intestinal flora on prostate cancer risk. Asian Pac J. Cancer Prev. 14, 1–4. 10.7314/apjcp.2013.14.1.1 23534704

[B116] Tak Y. E. Kleinstiver B. P. Nuñez J. K. Hsu J. Y. Horng J. E. Gong J. (2017). Inducible and multiplex gene regulation using CRISPR-Cpf1-based transcription factors. Nat. Methods 14, 1163–1166. 10.1038/nmeth.4483 29083402 PMC5909187

[B117] Takada T. Kurakawa T. Tsuji H. Nomoto K. (2013). Fusicatenibacter saccharivorans gen. nov., sp. nov., isolated from human faeces. Int. J. Syst. Evol. Microbiol. 63, 3691–3696. 10.1099/ijs.0.045823-0 23625266

[B118] Tamura K. Stecher G. Kumar S. (2021). MEGA11: molecular evolutionary genetics analysis version 11. Mol. Biol. Evol. 38, 3022–3027. 10.1093/molbev/msab120 33892491 PMC8233496

[B119] Tolonen A. C. Beauchemin N. Bayne C. Li L. Tan J. Lee J. (2022). Synthetic glycans control gut microbiome structure and mitigate colitis in mice. Nat. Commun. 13, 1244. 10.1038/s41467-022-28856-x 35273143 PMC8913648

[B120] Tolonen A. C. Cerisy T. El-Sayyed H. Boutard M. Salanoubat M. Church G. M. (2015a). Fungal lysis by a soil bacterium fermenting cellulose. Environ. Microbiol. 17, 2618–2627. 10.1111/1462-2920.12495 24798076

[B121] Tolonen A. C. Chilaka A. C. Church G. M. (2009). Targeted gene inactivation in Clostridium phytofermentans shows that cellulose degradation requires the family 9 hydrolase Cphy3367. Mol. Microbiol. 74, 1300–1313. 10.1111/j.1365-2958.2009.06890.x 19775243 PMC2810439

[B122] Tolonen A. C. Haas W. Chilaka A. C. Aach J. Gygi S. P. Church G. M. (2011). Proteome-wide systems analysis of a cellulosic biofuel-producing microbe. Mol. Syst. Biol. 7, 461. 10.1038/msb.2010.116 21245846 PMC3049413

[B123] Tolonen A. C. Zuroff T. R. Ramya M. Boutard M. Cerisy T. Curtis W. R. (2015b). Physiology, genomics, and pathway engineering of an ethanol-tolerant strain of Clostridium phytofermentans. Appl. Environ. Microbiol. 81, 5440–5448. 10.1128/AEM.00619-15 26048945 PMC4510172

[B124] Tran M. H. Park H. Nobles C. L. Karunadharma P. Pan L. Zhong G. (2021). A more efficient CRISPR-Cas12a variant derived from Lachnospiraceae bacterium MA2020. Mol. Ther. Nucleic Acids 24, 40–53. 10.1016/j.omtn.2021.02.012 33738137 PMC7940699

[B125] Uchiyama J. Akiyama M. Hase K. Kumagai Y. Kim Y.-G. (2022). Gut microbiota reinforce host antioxidant capacity via the generation of reactive sulfur species. Cell Rep. 38, 110479. 10.1016/j.celrep.2022.110479 35263581

[B126] Udayappan S. Manneras-Holm L. Chaplin-Scott A. Belzer C. Herrema H. Dallinga-Thie G. M. (2016). Oral treatment with Eubacterium hallii improves insulin sensitivity in db/db mice. NPJ Biofilms Microbiomes 2, 16009. 10.1038/npjbiofilms.2016.9 28721246 PMC5515273

[B127] Vacca M. Celano G. Calabrese F. M. Portincasa P. Gobbetti M. De Angelis M. (2020). The controversial role of human gut Lachnospiraceae. Microorganisms 8, E573. 10.3390/microorganisms8040573 PMC7232163 32326636

[B128] Vedanta Biosciences, Inc (2023). Vedanta Biosciences VE303 program. https://www.vedantabio.com/pipeline/ve303.

[B129] Vera-Ponce de Leon A. Schneider M. G. Jahnes B. C. Sadowski V. Camuy-Vélez L. A. Duan J. (2022). Genetic drift and host-adaptive features likely underlie cladogenesis of insect-associated Lachnospiraceae. Genome Biol. Evol. 14, evac086. 10.1093/gbe/evac086 35679131 PMC9210297

[B130] Vital M. Howe A. C. Tiedje J. M. (2014). Revealing the bacterial butyrate synthesis pathways by analyzing (meta)genomic data. MBio 5, e00889. 10.1128/mBio.00889-14 24757212 PMC3994512

[B131] Wachenheim D. E. Patterson J. A. (1992). Anaerobic production of extracellular polysaccharide by Butyrivibrio fibrisolvens nyx. Appl. Environ. Microbiol. 58, 385–391. 10.1128/aem.58.1.385-391.1992 16348636 PMC195219

[B132] Walker A. C. Bhargava R. Vaziriyan-Sani A. S. Pourciau C. Donahue E. T. Dove A. S. (2021). Colonization of the *Caenorhabditis elegans* gut with human enteric bacterial pathogens leads to proteostasis disruption that is rescued by butyrate. PLoS Pathog. 17, e1009510. 10.1371/journal.ppat.1009510 33956916 PMC8101752

[B133] Warnick T. A. Methé B. A. Leschine S. B. (2002). Clostridium phytofermentans sp. nov., a cellulolytic mesophile from forest soil. Int. J. Syst. Evol. Microbiol. 52, 1155–1160. 10.1099/ijs.0.02125-0 12148621

[B134] Watanabe M. Kaku N. Ueki K. Ueki A. (2016). Falcatimonas natans gen. nov., sp. nov., a strictly anaerobic, amino-acid-decomposing bacterium isolated from a methanogenic reactor of cattle waste. Int. J. Syst. Evol. Microbiol. 66, 4639–4644. 10.1099/ijsem.0.001403 27506535

[B135] Whitman W. B. (2009). Systematic bacteriology. New York, NY, USA: Springer. 10.1007/978-0-387-68489-5

[B136] Wolin M. J. Miller T. L. Collins M. D. Lawson P. A. (2003). Formate-dependent growth and homoacetogenic fermentation by a bacterium from human feces: description of Bryantella formatexigens gen. nov., sp. nov. Appl. Environ. Microbiol. 69, 6321–6326. 10.1128/AEM.69.10.6321-6326.2003 14532100 PMC201199

[B137] Wu C. Xu W. Yu W. Li Z. Zhang Y. Zhang F. (2021). Strain-level screening of human gut microbes identifies Blautia producta as a novel anti-hyperlipidemic probiotic via the production of 12-methylmyristic acid. https://www.researchsquare.com/article/rs-989302/v1. 10.1080/19490976.2023.2228045 PMC10324434 37408362

[B138] Yamashita H. Fujisawa K. Ito E. Idei S. Kawaguchi N. Kimoto M. (2007). Improvement of obesity and glucose tolerance by acetate in Type 2 diabetic Otsuka Long-Evans Tokushima Fatty (OLETF) rats. Biosci. Biotechnol. Biochem. 71, 1236–1243. 10.1271/bbb.60668 17485860

[B139] Yang C. Guan L. Liu J. Wang J. (2015). Rumen fermentation and acetogen population changes in response to an exogenous acetogen TWA4 strain and *Saccharomyces cerevisiae* fermentation product. J. Zhejiang Univ. Sci. B 16, 709–719. 10.1631/jzus.B1500013 26238546 PMC4534548

[B140] Zhang J. Song L. Wang Y. Liu C. Zhang L. Zhu S. (2019). Beneficial effect of butyrate-producing Lachnospiraceae on stress-induced visceral hypersensitivity in rats. J. Gastroenterol. Hepatol. 34, 1368–1376. 10.1111/jgh.14536 30402954 PMC7379616

[B141] Zhang L. Li G. Zhang Y. Cheng Y. Roberts N. Glenn S. E. (2023). Boosting genome editing efficiency in human cells and plants with novel LbCas12a variants. Genome Biol. 24, 102. 10.1186/s13059-023-02929-6 37122009 PMC10150537

[B142] Zhou Y. Wei Y. Jiang L. Jiao X. Zhang Y. (2023). Anaerobic phloroglucinol degradation by Clostridium scatologenes. mBio 14, e0109923–23. 10.1128/mbio.01099-23 37341492 PMC10470551

[B143] Zuroff T. R. Barri Xiques S. Curtis W. R. (2013). Consortia-mediated bioprocessing of cellulose to ethanol with a symbiotic Clostridium phytofermentans/yeast co-culture. Biotechnol. Biofuels 6, 59. 10.1186/1754-6834-6-59 23628342 PMC3653780

